# Environmentally Friendly Masonry Mortar Blended with Fly Ash, Corn Cob Ash or Ceramic Waste Powder

**DOI:** 10.3390/ma16206725

**Published:** 2023-10-17

**Authors:** Slobodan Šupić, Mirjana Malešev, Vladan Pantić, Ivan Lukić, Vlastimir Radonjanin, Miloš Ognjanović, Gordana Broćeta

**Affiliations:** 1 Department of Civil Engineering and Geodesy, Faculty of Technical Sciences, University of Novi Sad, 21000 Novi Sad, Serbia; ssupic@uns.ac.rs (S.Š.); miram@uns.ac.rs (M.M.); pantic_vladan@uns.ac.rs (V.P.); radonv@uns.ac.rs (V.R.); 2 Vinča, Institute of Nuclear Sciences, University of Belgrade, 11000 Belgrade, Serbia; miloso@vin.bg.ac.rs; 3 Faculty of Architecture, Civil Engineering and Geodesy, University of Banja Luka, 78000 Banja Luka, Bosnia and Herzegovina; gordana.broceta@aggf.unibl.org

**Keywords:** fly ash, ceramic powder, corn cob ash, sustainable mortar, binder replacement

## Abstract

Implementing a circular approach through waste valorization in mortar production with environmentally efficient mix design is a viable pathway for relieving the ecological burden of greenhouse gas emissions, resource depletion and waste management. The main objective of this paper is to evaluate the feasibility of using fly ash (FA), corn cob ash (CCA), and ceramic waste powder (CWP) as supplementary cementitious materials (SCM) in cement–lime masonry mortars. As part of an extensive experimental study, twelve mortar mixtures were made: three reference and nine blended, with mixing ratios of 1:1:5, 1:0.7:4.2, and 1:1:4 ((cement + SCM)/lime/sand), by volume. The examined properties include workability, compressive and flexural strengths, dry bulk density, capillary water absorption, adhesive bond strength, and water vapor permeability. The compressive and flexural strengths of tested mortars were notably impaired, with reductions of up to 60%, while the capillary water absorption coefficient rose by 100% compared to the reference values. The adhesive bond strength of some blended mortars exceeded the strength of the reference mortars. Nevertheless, all blended mortars fulfilled the requirements for general-purpose mortars, while the majority met the criteria for structural masonry applications. In addition, a performance-based index and weighting triangle were used for the comparison and ranking of all analyzed mortar mixtures. The findings of this study may herald a novel use of FA, CCA, and CWP as more eco-friendly binding materials in contemporary construction leading to the reduction in the process’s carbon footprint, the improvement in cost efficiency, and the mitigation of the detrimental environmental impact of waste disposal.

## 1. Introduction

With the continuous advancement of urbanization all around the globe in recent decades, the amount of industrial and agricultural solid waste has also increased dramatically. The proper utilization of these wastes rather than landfilling has become one of the most serious challenges for sustainable urban development.

According to reports, more than 900 million tons of coal fly ash (FA) are produced annually around the world; China produces about 580 million tons, India produces about 169.25 million tons, the United States produces about 43.5 million tons, Australia produces about 14 million tons, and Serbia produces about 7 million tons. It is estimated that 300 million tons of FA have already been deposited over a dumping area larger than 1500 ha in Serbia [1,2]. Unfortunately, the utilization rate of FA is not as effective as its production, currently standing at 53.5% globally and varying very widely, from nearly 100% in Japan to only a few percent in other countries (amounting up to 3% in Serbia). As a result, FA is most commonly disposed of in landfills, causing a slew of waste disposal and health risks.

On a similar note, another solid waste that must be considered for environmental protection is agricultural waste, which is producing in growing quantities annually and poses a severe threat to the environment when disposed of in landfills. Harvest residues can be transformed into an energy carrier and may be utilized as fuel during thermal, chemical, and microbiological processes [3]. However, a notable shift from the fossil fuel to the renewable biomass industry (developing bio-based processes, opening up new markets and creating industrial technologies) is required to establish a sustainable bioeconomy. Novel investigations, conducted by [4], reveal an encouraging possibility of employing biomass for CO_2_ storage. Agricultural biomass, i.e., harvest residues, represents a dominant renewable energy source (RES) in Vojvodina province in Serbia, accounting for 1.67 tons of oil equivalent. Although this resource is scarcely utilized (approx. 2%), considerable amounts of biomass ash (BA) are produced as waste products during the combustion of harvest residues, roughly estimated at 5000 tons per year [5]. National biomass ash production data are not routinely published, and the yearly output is changing rapidly as many countries strive to shift to a low-carbon economy. As a result, estimating the global production of ash from sustainable fuels is difficult. Globally, it was estimated that 7 billion tons (Gt) of biomass was burned annually for energy production in 2013, generating about 480 million tons of ash [6].

Apart from FA and BA, ceramic waste is a valuable resource in Vojvodina. It can be generated during the demolition of masonry facilities (demolition waste), and the construction of new buildings (construction waste), or it can originate from poor craftsmanship and production errors of clay-based elements (industrial waste). Every year, the total amount of waste produced by ceramic plants makes up about 3–7% of their entire final output. It is estimated that the ceramics industry in Vojvodina produces 6000 tons of ceramic waste annually [7]. In comparison, the amount of ceramic waste produced daily in Goa (India’s smallest state) is 30 million tons, while around 13 million tons of waste ceramics are discarded every year in China [8]. In addition, as reported in [9], the world’s output of sanitary ceramics reached 7.7 million tons in 2014, with almost 50% being produced in Asia, followed by the European Union, which accounted for 11.9% of the global production (0.91 million tons). As this type of waste is accumulating constantly, it is important to find a viable solution for this issue. Therefore, the utilization of ceramic waste in mortar or concrete manufacturing, as a pozzolanic mineral additive to replace cement, can be seen as a feasible option for contributing to this solution.

Residential buildings in the Serbian province of Vojvodina, whether single-family or multi-family, traditionally have brick walls. Various types of mineral-origin mortar are commonly used during the construction of masonry structures. According to earlier assessments [10], 198,000 tons of mortar is consumed annually in Vojvodina for masonry and plastering purposes in newly constructed apartments.

Given that the common mixing ratio of cement, hydrated lime, and sand in masonry mortar is 1:2:5, it takes approximately 230 kg of cement and 270 kg of hydrated lime to make 1 m^3^ of mortar. Taking the estimated yearly mortar requirements into account, the consumption of cement and hydrated lime amounts to approximately 27,000 tons and 31,500 tons, respectively. Cement and hydrated lime are produced using technological processes that rely heavily on fossil fuels and non-renewable natural resources. For instance, one ton of cement requires around 1.5 t of raw materials, while 1 ton of lime requires 1.4 t of limestone for their production.

Apart from natural resource consumption, a large amount of CO_2_ is discharged into the environment due to the thermal treatment of raw materials in these processes. According to estimates, the production of 1 t of cement releases approx. 0.6 t of CO_2_, while 1 t of lime generates an even higher amount, evaluated at 0.8 t of CO_2_ [11]. In light of these data, it is simple to calculate that 40.5 thousand tons of limestone and clay are needed annually to produce cement, and 44.1 thousand tons of limestone is needed to produce lime, while approx. 41,500 tons of CO_2_ is released into the atmosphere.

Finally, it should be noted that conventional masonry mortars made of cement and lime are not ecologically friendly in 21st-century civil engineering practice, since their production does not adhere to the fundamentals of sustainable development. Therefore, using locally accessible industrial and agricultural by-products could be a viable option to create new, alternative forms of greener masonry mortars.

In recent years, extensive research has been conducted on the application of fly ash, corn cob ash (CCA) and ceramic waste powder (CWP) as pozzolanic cement replacing materials in cement-based composites. Many studies have demonstrated that using these by-products can enhance the microstructure and improve the durability properties of cement-based mortar and concrete [12,13,14,15,16,17,18,19]. Contrastingly, the application of agricultural and industrial waste in masonry mortars has been investigated to a limited extent. Ebrahimi et al. [20] examined the effect of CWP as a binder alternative in cement and lime-based masonry mortars (up to 80%) to create an eco-friendly mortar. According to their findings, the compressive and tensile strengths of cement mortars were slightly increased when up to 50% of the cement was replaced with CWP. The strengths of hydrated lime mortars were significantly increased (up to ten times) with an increasing substitution rate up to 70%, identifying CWP as a good sustainable choice for cement replacement. Nayaka et al. [21] explored the viability of using palm oil fuel ash (POCWP) as a cement substitute up to 80% in a mixing ratio of 1:0.5:4.5 (cement/lime/sand). They observed that POCWP may greatly lower the carbon footprint by around 32% compared to the conventional mortar while maintaining the mechanical and fresh performances of mortar with the inclusion of POCWP up to 50% as a supplementary cementitious material (SCM). Another study [22] by the same group of authors found that 40% of cement replaced by POCWP appears to produce the most reliable masonry mortar (1:0.5:4.5) in terms of durability. An investigation on masonry mortar (1:2:4) blended with FA and wheat straw ash (WSA) showed that these by-products can efficiently replace cement up to a level of 30% without reducing its strength to a larger extent [23]. On the other hand, the authors outlined that cement–lime mortars blended with 50% of granulated blast furnace slag (GBFS) as cement replacement outperformed the reference mortar in terms of strength. The available literature does not provide information on CCA application in masonry; hence, this by-product in Serbia is here tested for possible application in masonry mortars for the first time, which demonstrates the innovative character of this research.

It is obvious that industrial and agricultural waste materials could be viable SCMs, particularly in terms of sustainable development. However, the effects of using various SCMs as a binder replacement in different mixing ratios in masonry mortars have not been thoroughly examined yet. The ceramic waste powder as a pozzolanic material has only been briefly studied, while there is no published research on the cement–lime masonry mortar incorporating corn cob ash as an SCM. Such research is especially impactful for geographical regions around the world that are subjected to anthropogenic pollution. With the aforesaid objections in mind, the authors of this paper evaluated the influence of locally sourced agricultural and industrial wastes—corn cob ash, fly ash and ceramic waste powder—on different properties of masonry mortars, including the compressive and flexural strength, dry bulk density, capillary water absorption, adhesive bond strength, and water vapor permeability. To clarify and bolster the results of these tests, microstructural (XRD) investigations were also performed. Furthermore, the carbon emissions and cost efficiency of mortars blended with selected wastes are also elaborated to assess environmental sustainability. The findings of this study introduce the compelling utilization of FA, CCA, and CWP as more environmentally acceptable binder materials in contemporary construction, leading to a reduction in the carbon footprint of the construction process, the improvement in cost efficiency, and the mitigation of the detrimental environmental impact of waste landfilling.

## 2. Materials and Methods

### 2.1. Materials

The material used was ordinary Portland cement (OPC), produced by the Lafarge cement plant in Beočin, Serbia. The cement is characterized by a density of 3.1 g/cm^3^ and the Blaine fineness of 4.000 cm^2^/g.

Fly ash, corn cob ash, and ceramic waste powder were used as cement replacement materials in the cement–lime masonry mortars.

**Figure 1:**
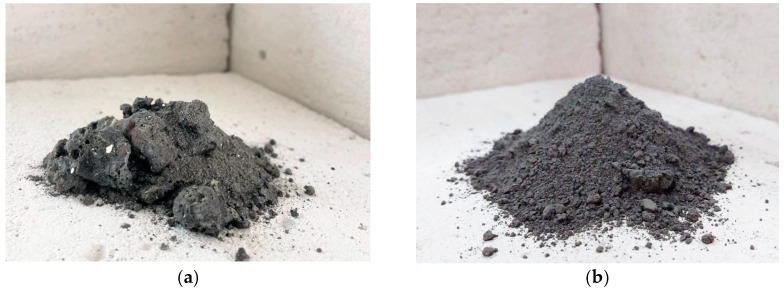
(**a**) Corn cob ash, before grinding. (**b**) Corn cob ash, after grinding.

Corn cob ash (CCA) was collected from ALMEX-IPOK in Zrenjanin, Serbia. This starch factory uses biomass waste—corn cobs—as an energy source and generates substantial amounts of CCA as a by-product. To obtain a material with a satisfactory level of fineness, the collected ash sample was ground in a laboratory ball mill (Figure 1).

**Figure 2:**
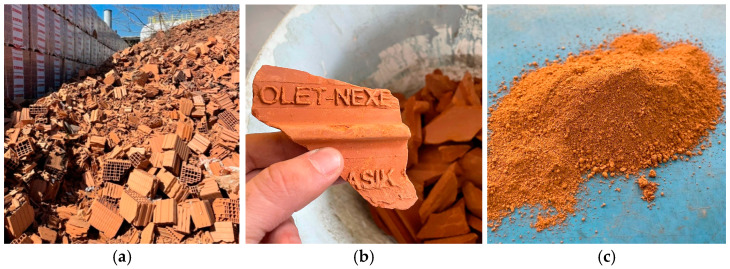
(**a**) Ceramic manufacturing waste. (**b**) Crushed clay blocks. (**c**) Ceramic waste powder, after grinding.

The ceramic waste powder (CWP) was produced from ceramic manufacturing waste, consisting of damaged clay hollow blocks discarded in the production facility NEXE—Stražilovo in Petrovaradin, Serbia. These elements were firstly roughly crushed and then finely ground in a lab ball mill up to the appropriate level of fineness (Figure 2).

Fly ash (FA) was provided by the thermal power plant Nikola Tesla B in Obrenovac, Serbia. As the fineness of FA was satisfying, no additional mechanical processing was necessary.

The results of testing the chemical composition of SCMs are summarized in Table 1.

The principal oxides of FA and CWP are SiO_2_ and Al_2_O_3_, which together make up over 75 wt% of the total oxides. It can also be seen from the table above that these materials are rich in Fe_2_O_3_, which adds up to 80 wt% of the oxides in their chemical compositions. CCA primarily consists of 45.76% SiO_2_, 14.08% CaO and 13.10% K_2_O. The total alkali content of CCA (Na_2_O + 0.658 K_2_O) is estimated as 8.62%, exceeding the permissible value of 5% prescribed in EN 450-1 [23]. Hence, the detrimental effects of a potential alkali–silica reaction (ASR) should be taken into consideration while analyzing the durability aspects of mortars blended with this material.

Based on the total content of important oxides, SiO_2_ + Al_2_O_3_ + Fe_2_O_3_ (min. 70%, SRPS EN 450-1), and the reactive SiO_2_ content (min. 25%, SRPS EN 450-1), FA and CWP could be classified under pozzolana. CCA satisfies the latter criterion, which is likely to result in the satisfactory pozzolanic activity of this SCM.

The physical properties of cementitious materials are shown in Table 2.

The specific surface area of SCMs is slightly higher than that of cement, which could be beneficial from the perspective of their reactivity and packing capacity.

As a result of sufficient amorphous silica content and a satisfactory level of fineness, all tested materials displayed positive pozzolanicity, whereas FA and CWP showed a pozzolanic activity of Class 10, while CCA demonstrated a pozzolanic activity of Class 5.

The FA and CWP specimens met the requirements regarding the activity index, while CCA fulfilled not only the prescribed criteria but also attained compressive strength higher than that of the reference cement specimen. This is probably due to the enhanced compactness of the mortar mix blended with finer biomass ash particles.

All tested materials met the other requirements regarding physical properties from the applicable standards, as indicated in Table 2.

The river-derived sand was employed as a fine aggregate for mortar production. Its specific gravity and fineness modulus were determined to be 2.3 g/cm^3^ and 0.97, respectively.

Masonry mortar was produced using tap water. Aiming to produce the necessary workability of masonry mortar (175 ± 10 mm), as recommended by SRPS EN 1015-2 [24], the water-to-binder ratio (w/b) was adjusted.

### 2.2. Methods

The chemical composition of raw materials was assessed according to EN 196-2 [25] and ISO 29581-2 [26].

The pozzolanic activity was analyzed for the specimens prepared in accordance with the instructions provided in SRPS B.C1.018 [27]. Mortar specimens were produced with SCM, slaked lime and CEN sand, with the following mass proportions: m_sl_:m_SCM_:m_qs_ = 1:2:9 and a water-to-binder ratio of 0.6 (where m_sl_—mass of slaked lime; m_SCM_—mass of SCM; m_qs_—mass of CEN sand).

The activity index was tested as described in EN 450-1 [28]. In line with EN 196-1’s provisions, standard mortar specimens were prepared, and the compressive strength was measured [29].

Mechanical characteristics (compressive and flexural strength) were tested as instructed by EN 998-2 [30] and EN 1015-11 [31]. The workability of fresh mortar (flow value) was determined in accordance with EN 1015-3 [32]. Using the specifications provided in EN 1015-18, the water absorption coefficient triggered by the capillary action of hardened mortar was calculated [33]. The mortar’s dry bulk density was specified as explained in EN 1015-10 [34]. The adhesive strength of hardened mortars on substrates was assessed in accordance with the procedure described in EN 1015-12 [35]. The water vapor permeability of hardened masonry mortars was determined using EN 1015-19 [36].

**Figure 3:**
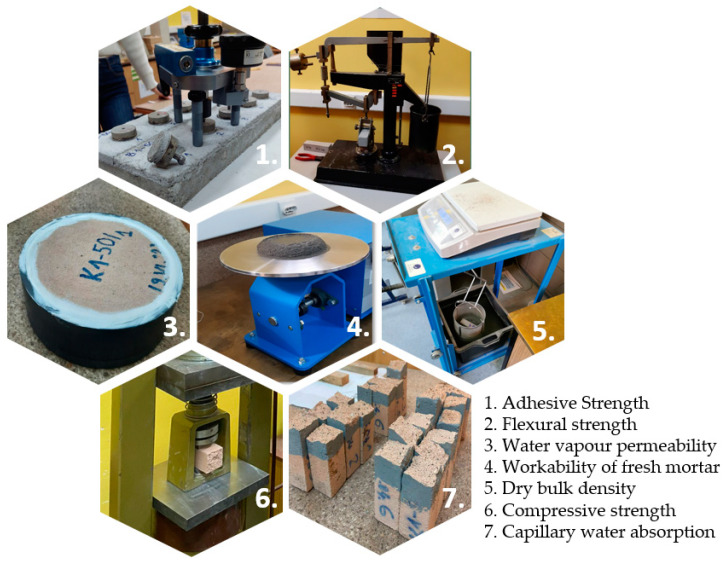
Preparation and testing of mortar mixtures.

Preparation and testing of different properties of mortar mixtures are illustrated on Figure 3.

### 2.3. Mixing and Proportioning of Mortars

Twelve different mortar combinations were used in the experimental investigation. Predicated on the results of the laboratory trial conducted prior to the study, the following volume mixing ratios of components of reference cement–lime mortars were utilized: 1:1:5, 1:0.7:4.2, and 1:1:4 (cement/lime/sand). In the remaining nine mixtures, 50% of cement was replaced by FA, CCA, or CWP for each mixing ratio by volume. Based on the designed workability, the appropriate amount of water was determined. Table 3 shows the labels and quantities of component materials for each masonry mortar.

The materials—cement, hydrated lime, SCMs and sand—were mixed for about 3 min using a mortar mixer to obtain a homogenous mixture. First, homogenization of the lime and cementitious powder was performed. The binder mix was then poured into a water-filled bowl, while the sand was automatically added at a constant rate during the first mixing phase in the mixer. The storage and curing conditions of different mortar samples were adjusted in accordance with relevant standards and the samples were tested at the appropriate age.

## 3. Test Results and Discussion

### 3.1. Mortar Properties

#### 3.1.1. Workability of Fresh Mortar

**Figure 4:**
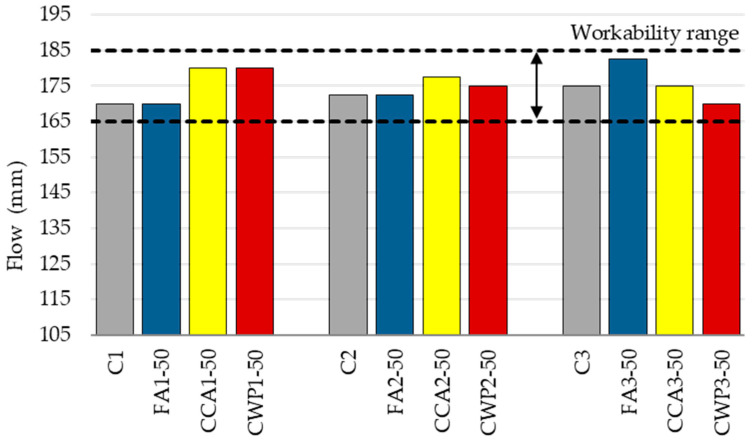
Flow values of fresh mortar.

The influence of the water-to-binder ratio (w/b) on the necessary workability (175 ± 10 mm) was determined on the flow table. The flow values are given in Figure 4.

The results reveal that when an SCM is added to the mortar mixture, it becomes less workable, requiring additional water to make the blends more feasible. As a result, w/b rises with the increase in SCM content to ensure that the appropriate workability can be attained in the blended mixtures.

Some authors have found that the workability of cement-based composites blended with corn cob ash could be impaired due to the angularity and sharp edges of ash particles [37]. In our study, the flow of the reference mortar (C1) decreased from 175 mm to 140 mm when 50% of cement was substituted with CCA, representing a decline of 20%. The finding is in agreement with the experimental studies conducted by Olafusi et al. [38] and Adesanya et al. [39].

Similarly, as the CWP content increased, the flow of the mortars decreased. The flow of the reference mortar (C1) dropped by 22.2% upon 50% replacement of PC with CWP. The porous nature of ceramic material, which increases its absorption rate, could be the reason for the reduction in flow values of the mortar blended with CWP. This finding is consistent with the study of Torres at al. [40]. Additionally, it was observed that ceramic particles tend to agglomerate during compaction. In this light, Zhang et al. [41] also mentioned that rapid agglomeration leads to a lower flow value. Rahhal et al. [42] analyzed the morphologies of ceramic particles via SEM and noted that “particles mostly had an irregular shape and partially layered microstructure with a high percentage of fineness”.

The workability of FA-based composites depends mainly on the shape of the FA particles. A number of authors mentioned that FA regularly contains smooth spherical fine particles that act as plasticizers and ball bearings that release trapped water within cement particles, reduce friction between aggregate grains and improve workability [43,44]. In contrast, according to the findings of Fantu et al. [45], substituting more than 10% of cement with FA could reduce the workability of cement-based composites. In addition, some authors stated that the irregular shape and porous nature of certain particles of FA may contribute to a slight workability reduction, regardless of the substitution level [46]. In this study, it was noted that FA-based blends need more water to maintain the required flow value.

Furthermore, the higher level of fineness, i.e., the specific surface area, plays its dominating role when SCM content rises, which in turn increases the water demand, regardless of the SCM type.

Meanwhile, as the sand/binder ratio decreases, the content of the binder component in the mortar increases, which improves the fluidity of the mixture. As a result, mortars with the mixing ratio of 1:1:4 displayed the lowest water demand and are characterized by the lowest w/b. This is likely to have a significant impact on all of the properties of masonry mortars.

#### 3.1.2. X-ray Powder Diffraction Technique Analysis

**Figure 5:**
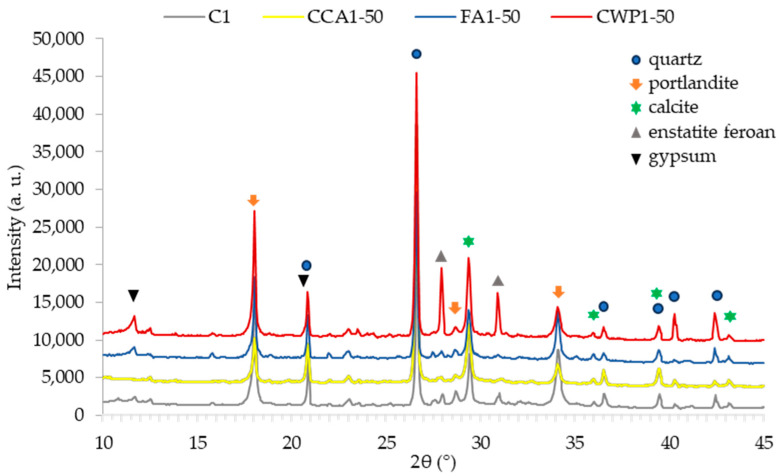
XRD patterns of mortars.

The XRD patterns collected on mortars, as displayed in Figure 4, were utilized for the evaluation of the mineralogical composition as well as for crystallinity studies. As seen from Figure 5, an amorphous phase cannot be observed in the XRD patterns of mortars. The mineralogical composition was determined by comparing the collected XRD patterns with the Inorganic Crystal Structure Database (ICSD) data. All of the investigated mortars consist mainly of quartz, portlandite, and calcite. Other minerals discovered include gypsum and enstatite ferroan. The presence of enstatite ferroan (Magnesium Iron Silicate) in a higher percentage was detected in CWP1-50. This is due to the fact that the used ceramic waste contained this mineral that could be formed from kaolinite at temperatures above 800 °C [47].

#### 3.1.3. Flexural Strength of Mortar

**Figure 6:**
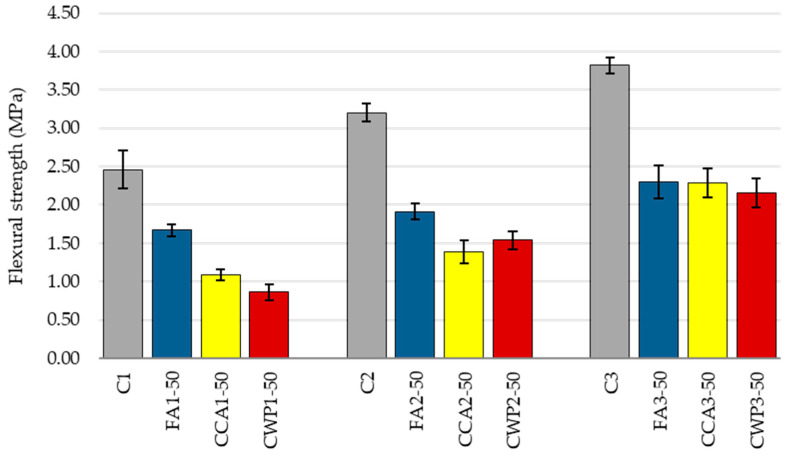
The flexural strength of mortars.

Figure 6 illustrates the variation trend of flexural strengths of mortar with the varying mixing ratios.

Cement/lime and sand/binder ratios influence the mechanical properties of concrete to a great extent. As expected, flexural strength rises with the increase in the cement/lime ratio and further increases with the decrease in the sand/binder ratio, which is, once again, associated with the corresponding w/b [48]. When compared to the reference mix (C1), the flexural strength of C2 improved by 30% as the w/b ratio dropped from 1.15 to 1.05, while the strength of C3 rose by 55% as the w/b ratio decreased to 0.9. Similar patterns were registered for SCM-blended mortars.

The use of the chosen SCMs, as cement replacing materials, caused a sharp flexural strength decrease, regardless of the mixing ratio. At the age of 28 days, FA1, CCA1 and CWP1 mixes attained about 68%, 44% and 35% of the equivalent control flexural strength, respectively. This remarkable strength reduction can be attributed to (1) the excess water provided for the workability adjustment, i.e., increased w/b, and (2) the dilution effect. As far as other mixing ratios are concerned, similar strength declines were observed, with slight changes in the strengths of SCM-blended mortars.

It should be noted that, unlike the workability, the adhesive and compressive strength, the flexural strength is not of prime importance for masonry applications.

#### 3.1.4. Compressive Strength of Mortar and Achieved Class

**Figure 7:**
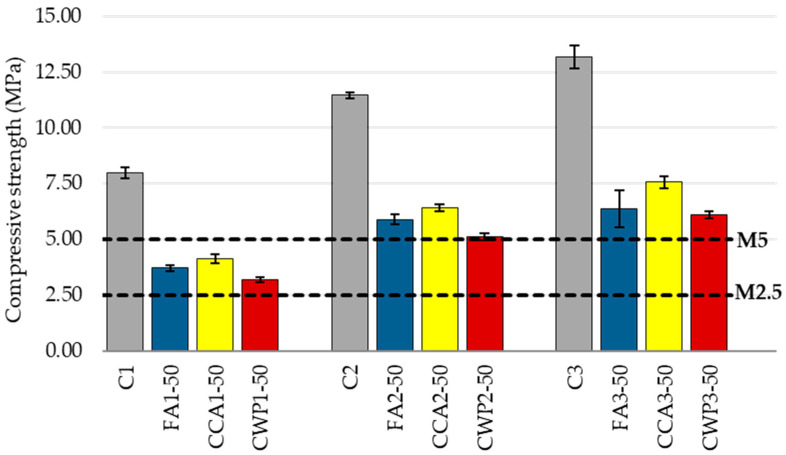
The compressive strength of mortars.

The compressive strength results follow a pattern similar to that of the flexural strength tests. In general, the trend of the compressive strength reveals that the addition of SCMs reduces the strength, regardless of the SCM type and mortar constituents’ volume proportion. As shown in Figure 7, the inclusion of 50% of FA, CCA, or CWP reduced the compressive strength by about 54%, 48%, and 60%, respectively, compared to the reference mix C1. A similar trend was observed for the remaining mixing ratios. The lower hydration activity of SCMs, i.e., the dilution effect, could be the reason for the decrease in compressive strength. This finding is in agreement with the experimental studies conducted by Nayaka et al. [21], Ohemeng et al. [49] and Lertwattanaruk et al. [50]. On a similar note, Nayaka et al. [22] stated that biomass ash, as an SCM, decreases the heat evolution rate and thus delays the hydration in the masonry mortar at an early age.

On a more important note, w/b is also an essential factor influencing the mechanical performance of cement-based composites. As the mortar blends significantly differ from the control specimens in this regard, the effect of increased w/b on the mechanical properties of environmentally friendly masonry mortars was anticipated. Higher w/b induced greater total and capillary porosity of mortar blends and, consequently, decreased the number of hydration products, leading to a decline in compressive strength.

Though negligible variations were noticed among the compressive strengths of mortars modified with SCMs, CCA blends displayed a compressive strength higher than that of the CWP and FA blends, which can be ascribed to (1) the higher fineness of this type of SCM, (2) the higher activity index, and (3) the w/b lower than that of the other two blends.

Considering the mortars with the other mixing ratios, as expected, a strength enhancement was observed for all mixes. For instance, the compressive strength of FA2, CCA2 and CWP2 mixes rose by 59%, 56% and 60%, while the strengths of FA3, CCA3 and CWP3 blends grew by 72%, 84% and 91% in relation to the corresponding strengths of mortars with the ratio 1:1:5.

Masonry mortars are categorized into classes depending on their mean compressive strength, as defined by EN 998-2. Eurocode 6 and Eurocode 8 specify a minimum compressive strength of 5MPa for masonry mortars for load-bearing structures, i.e., Class 5. Table 4 shows the average compressive strength and attained class of each mortar.

As displayed in the table above, all mortar blends with mixing ratios of 1:0.7:4.2 and 1:1:4 meet the criteria for masonry mortar for structural applications, while the blended mortars with the mixing ratio of 1:1:5 achieved the class of 2.5 and can thus be utilized successfully for masonry applications for non-load-bearing elements (such as infill and partition walls).

#### 3.1.5. Dry Bulk Density of Mortar

**Figure 8:**
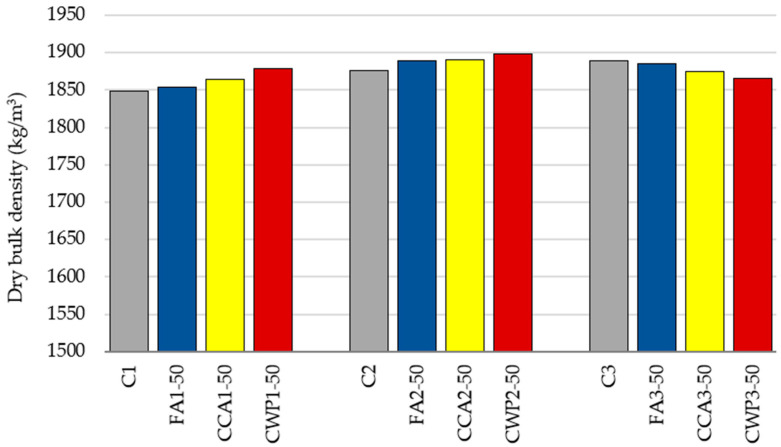
Dry bulk density of mortars.

The dry bulk density of the hardened mortar is depicted in Figure 8.

Due to the lower specific gravity of SCMs compared to cement, the density of the blended mortars with the mixing ratio of 1:1:4 decreases with the cement replacement as previously documented [18,19]. However, the densities of FA, CCA and CWP mortars fell by just 1% in comparison to the reference value, indicating the same range of the bulk density.

For the masonry mortars with the remaining mixing ratios, the dry bulk density of SCM-modified mortars is slightly increased by the cement substitution. This could be ascribed to the enhanced packing capacity of mixtures blended with finer particles and a greater quantity of sand. The small particles of SCMs act as a filler material, inserting themselves into the voids of the sand and Portland cement and contributing to the increased bulk density of the mortar. However, as the variations in bulk densities are less than 2%, it can be concluded that replacing cement with the selected SCMs has a negligible impact on this property of masonry mortar.

#### 3.1.6. Capillary Water Absorption

Water absorption, which indirectly expresses capillary porosity, is one of the elements determining the durability of cement-based composites.

**Figure 9:**
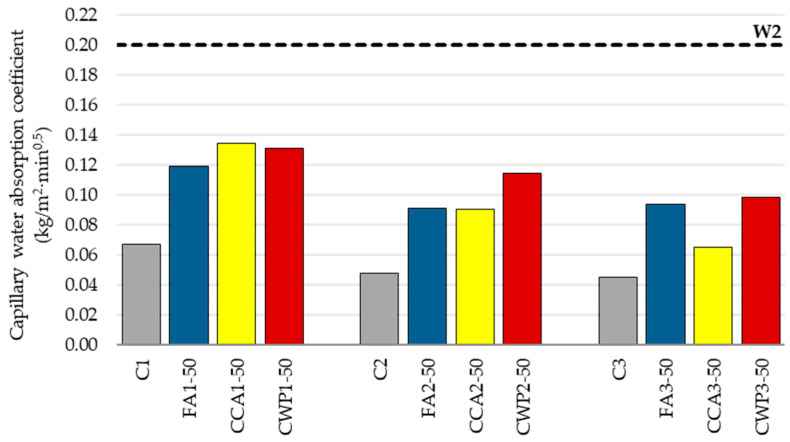
Capillary water absorption coefficients of hardened mortars.

The obtained findings demonstrate that, regardless of the mixing ratio, the mortar water absorption rose significantly with the cement substitution (Figure 9). When compared with the reference mortar with a 1:1:5 mixing ratio, FA-, CCA-, and CWP-modified mortars exhibited higher capillary water absorption coefficients by 78%, 101%, and 95%, respectively. This behavior is a consequence of the higher w/b and dilution effect, i.e., greater capillarity of blended masonry mortars.

One of the important aspects of cement-based materials, impacting the pore structure and capillary water absorption properties, is the water-to-binder ratio. As already stated above, all SCM-modified mortars were produced with an additional amount of water, aiming to satisfy the required workability. Correspondingly, using a higher w/b ratio, the capillary porosity of the mortar mixtures rose as the SCM content increased, resulting in a higher absorption coefficient.

Higher absorption resulted from the diluting effect, which caused the effective w/b to increase as cement concentration decreased (Table 5). Appropriately, all blended mortars showed greater capillary water absorption capacity compared to the corresponding cement–lime mortars. This finding can be explained by the fact that the total porosity of mortar rose, increasing its capability to accommodate water. This, in turn, enhanced the cement mixes’ capacity to absorb water. Effective w/b ratios were computed following the k-value concept, established for fly ash, in accordance with EN 206-1 [51,52].

It was reported by a number of authors (Saha et al. [53], Milović et al. [54]) that the permeability coefficient may be reduced as a consequence of the enhanced reaction between reactive silica in pozzolana and the products liberated during the hydration process, as the additional CSH gel fills the pores and densifies the structure of the mix. However, due to the prolonged pozzolanic reaction of SCMs at this early stage, a lower amount of hydration products is generated up to the age of 28 days, resulting in a decrease in active binder content and increase in water absorption of SCM-blended mortars.

Moreover, densification of the composite may occur as a consequence of a higher level of fineness of its constituents. Cordeiro et al. [55] also confirmed that the decrease in capillarity of the finely ground biomass ash-based mortars is directly linked to the filler effect provided by the ultrafine particles of the ash, which improves the packing behavior of the mix. Nevertheless, in this study, the increased w/b has dominance over the initially progressed pozzolanic response of the selected SCMs and the insufficient level of fineness, i.e., deficient packing capacity, of the blended mortar.

Much the same pattern was detected for the other mixing ratios. As a result of the higher fineness of corn cob ash, CCA-modified mortar with the ratio 1:1:4 displayed a lower absorption coefficient in relation to the other blended mortars of this series.

Based on the computed water absorption coefficient at the age of 28 days, masonry mortars can be categorized, as recommended by EN 998-2. As all mortars satisfy the criterion for the optimal W2 category (<0.2 kg/m^2^min^0.5^), it can be stated that the substitution of cement with the selected SCMs does not jeopardize this property of mortar to a more significant extent.

#### 3.1.7. Adhesive Strength of Mortar

The adhesive strength is one of the main requirements for masonry mortar, as the lack or loss of adhesion impairs the integrity and useful life of buildings, influencing the habitability, thermo-acoustic comfort, and sealing against atmospheric effects. The capacity of the mortar to retain water, its consistency, air content, and its mechanical strength are all elements that affect the mortar’s ability to adhere to surfaces.

Moreover, adhesive strength is affected by surface preparation, such as the removal of dirt that may deteriorate adhesion, as well as the substrate’s mechanical strength, absorption properties and porosity.

**Figure 10:**
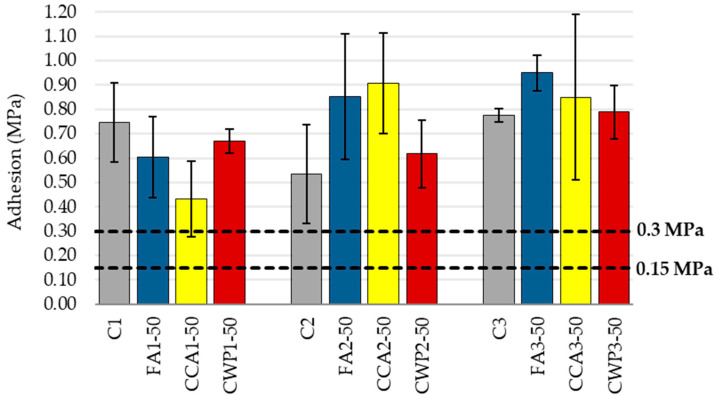
Adhesive strength of mortars.

Similarly to the compressive strength variations, the pull-off strengths dropped as SCM contents rose in the 1:1:5 mortar series (Figure 10). The registered strength decreases for the FA, CCA and CWP blends were 19%, 42% and 9%, respectively. As noted earlier, this finding can be ascribed to a dilution effect that reduces the early strength development and bond formations between the mortar and substrate. It is worth mentioning that the failure took place between the applied mortars and substrate, indicating that the interface is the weakest layer in the system.

Due to its porous structure, ceramic waste powder is inferred to retain water within mortar, thus contributing to bond strength. It prevents the migration of water from the mortar to the base, enabling longer binding processes of the CWP-modified mortar. This is why, despite the early stage of a pozzolanic reaction and poor mechanical properties, the adhesive strength of CWP-based mortars is on par with the strength of the reference specimens for each mixing ratio. This effect is consistent with previous research on this topic [56].

A further element that helps to strengthen the adhesive strength of mortars is an improvement in SCM particle dispersion. For example, Dvorkin et al. [57] investigated the effects of varied water–cement ratios, levels of fineness and the ratios of cement–ash binder to sand, finding that regrinding ash to a specific surface area increases the adhesive strength of the mortar. Finer particles of SCM-blended mixes might improve the adhesion of the mortar owing to a smaller number of voids present in between grains. This may be the reason why the adhesive strength ratios changed when FA and CCA were added to masonry mortars in the following mixing proportions: 1:0.7:4.2 and 1:1:4. In these mortar series, the adhesive strength of FA- and CCA-based mortars exceeded the strength of the corresponding reference specimens.

The prescribed minimum value in EN 998-1 for masonry mortars used in rendering or plastering is 0.3 MPa, while this value required by EN 998-2 is 0.15 Mpa.

All mortar combinations met the criteria for both plastering and masonry applications. The EN 1015-12 specification classifies the possible fracture patterns into three categories: (a) adhesion fracture: a fracture at the mortar–substrate interface, the test value of which is equivalent to the adhesive strength; (b) cohesion fracture: a fracture in the mortar itself, where the adhesive bond is stronger than the test value; (c) cohesion fracture: a fracture in the substrate material, where the adhesive bond is stronger than the test value. Recorded fracture patterns of masonry mortars are listed in following Table 6.

The adhesion to the substrate can be considered acceptable for all the mortars, considering the limit values defined in relevant regulations, especially when the cohesive fracture pattern within the mortar is considered rather than an adhesive pattern between the mortar and substrate for specific mortar types.

#### 3.1.8. Water Vapor Permeability

The water vapor diffusion properties of mortars are important in construction to manage moisture and prevent issues such as condensation, mold growth, and damage to building structures.

Table 7 displays the results for water vapor permeability and water vapor resistance factor for mortars with the mixing ratio of 1:1:5.

Water vapor permeability is primarily determined by matrix porosity as well as pore size and distribution [58,59,60]. It can be noted that the blended mortars have lower water vapor permeability than the reference mortar. Porosity and permeability have an inverse relationship when compared to the results of capillary absorption analysis. As the blended mortars have a higher fineness of binders, it is envisaged that the volume of pores related with vapor permeability will be lowered.

All mortars have water vapor resistance values in the range of 3.4–5.2. EN 998-1 [61] does not define limit values for masonry mortars for general purposes, while EN 12524 specifies a value of 6 for water vapor resistance for masonry mortars tested with the wet cup method.

### 3.2. Environmental Impact

As a result of direct emission from the limestone calcination and indirect emission from the combustion of fossil fuel, cement is labeled as a major contributor to carbon dioxide (CO_2_) emissions, estimated at 5–8% worldwide. According to projections, this trend is expected to increase to 27% by the year 2050 [62], owing to an ongoing increase in demand for new buildings brought on by the rapid growth of urban areas. Aiming to mitigate this projected scenario, researchers all around the globe have been developing a series of strategies and innovations to reduce CO_2_ emissions. One of the approaches is the deployment of environmentally friendly materials in the development of agro-industrial waste-based cement composites. Over the years, the environmental benefits of using agricultural and industrial by-products as SCMs have been extensively researched [63,64,65,66], resulting in lower global warming potential and greenhouse gas emissions, the preservation of natural resources and waste reduction.

In this study, the CO_2_ emission factors were used to gauge the eco-efficiency of SCM-blended mortars (Table 8). CO_2_ emissions, resulting from the mechanical processing of CWP and CCA, are low and may be regarded as negligible. Only CO_2_ emissions of OPC, FA, and sand (except for CO_2_ emissions produced during the transportation, handling, and placement processes) were considered. The estimation of CO_2_ emission factors for raw materials was taken from the previous studies conducted by Lukić et al. [67] and Hammond et al. [68].

**Figure 11:**
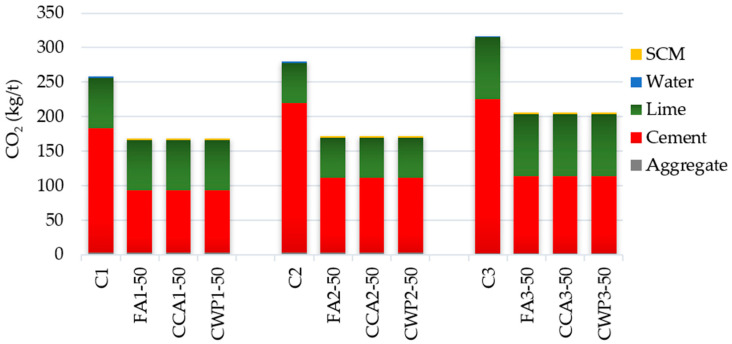
CO_2_ emissions of analyzed mortar mixtures.

The obtained emissions were multiplied by the volume of the raw ingredients to determine the total CO_2_ emissions of the masonry mortars. The results, illustrated in Figure 11, clearly show that replacing 50% of cement with the selected SCMs lowers the total CO_2_ emissions to a noteworthy extent. All blended mortars had similar emission drops of roughly 35%, 39%, and 35% for mortar mixing ratios of 1:1:5, 1:0.7:4.2, and 1:1:4, respectively, in relation to the reference mortar.

Hence, from the perspective of the carbon footprint, SCM-modified mortar has net benefits over reference masonry mortar, as it emits 35–40% less CO_2_ during its manufacturing process. Thus, a more sustainable composite with suitable properties may be developed by employing fly ash, corn cob ash, and ceramic waste powder as SCMs.

### 3.3. Cost Efficiency

Cost efficiency is one of the crucial parameters for assessing the sustainability of cement-based materials. The raw material unit costs were calculated using the Serbian raw material purchase price; the calculations did not include the costs associated with the transportation, handling, placement, and quality control. The unit costs of all component materials of the masonry mortars are listed in Table 9.

**Figure 12:**
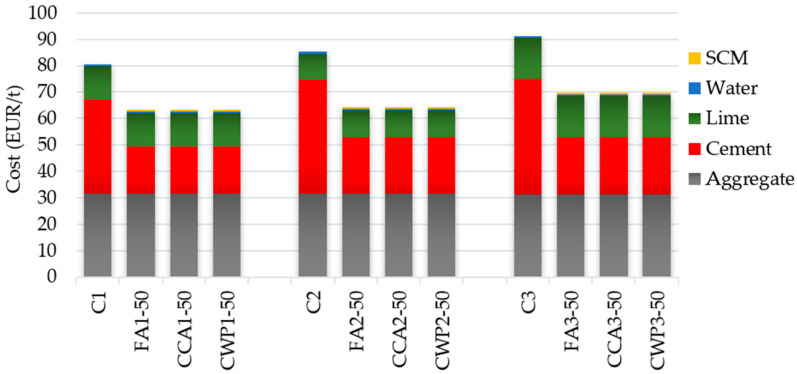
Cost price of analyzed mortar mixtures.

As the prices of the mortar do not change much when the type of aggregate is varied, only the prices of the binder components were considered for evaluating the costs of mortar mixtures. The inclusion of SCMs in various proportions in masonry mortar had a significant economic effect regardless of the employed mixing ratio. The blended masonry mortars were more economical than the reference ones, by 25%, 22% and 24% for mortar mixing ratios of 1:1:5, 1:0.7:4.2 and 1:1:4, respectively (Figure 12). Hence, the use of agricultural and industrial by-products in masonry mortars led to an improvement in the cost efficiency, which reflects the strong sustainability of the utilized SCMs as a resource.

## 4. Performance Index

The concept of the performance index (PI) is used as a quantitative value to describe the properties of masonry mortar blended with SCMs in relation to the conventional cement–lime masonry mortar [69]. Performance improves as the PI increases. Each examined parameter with the best result is assigned a numerical value of 1.0 (highlighted in Table 10). If the values of PI are lower than 1.0, mortars have an inferior performance compared to the optimal value.

The performance index for each type of tested mortar was established based on the compressive strength, flexural strength, capillary water absorption, adhesive strength, CO_2_ emission, and cost effectiveness.

It can be noted that the performance index declines with cement replacement considering the tested physical and mechanical properties, while it increases owing to the environmental and cost efficiency effects of SCM utilization. As a result, the total performance index of blended masonry mortar is slightly lower than that of the corresponding reference values. Reference mix C3 has a higher total PI than blended mortars, while, among the SCM-modified mortars, CCA3-50 has the highest total PI.

## 5. Weighting Triangle

A weighting triangle is an easy-to-understand graphical representation of three parameters influencing masonry mortar performance, which could support decision making processes in the composite selection. A weighting triangle can be used to represent any possible arrangement of relative weights for three specified parameters. A weighing combination is indicated by each point in the triangle. Every point in the triangle has relative weights that add up to 100%. The question of the weighting triangle is thus: what percentage of the total weight is given to each of the three parameters, and how much weight is acceptable for each parameter in the decision-making process?

In this study, we chose the following influencing parameters: (1) the compressive strength, (2) environmental impact (CO_2_ emissions), and (3) cost efficiency. Given that the achieved mortar class defines the possible application of masonry mortar, the compressive strength was selected as a key property, and an influencing parameter inside the weighting triangle. By assigning an equal significance to each influencing factor, the weights of these parameters were determined based on the previously calculated performance indexes and are listed in Table 11.

**Figure 13:**
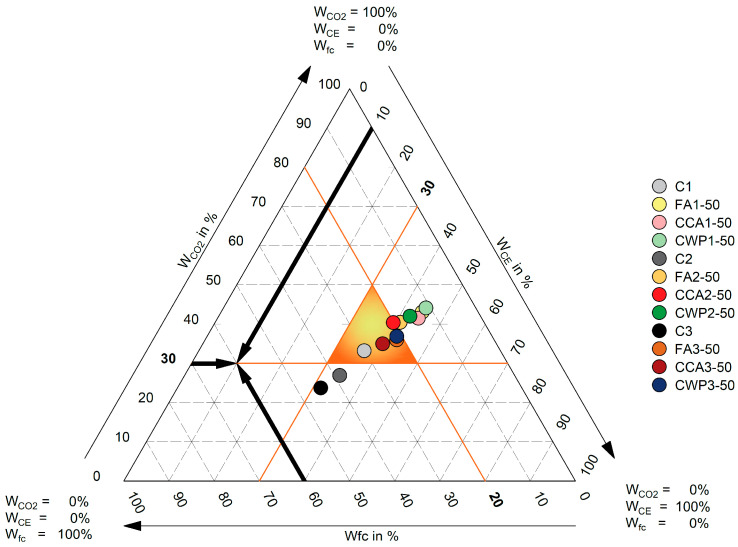
Weighting triangle.

A weighting triangle’s corners each denote a parameter’s 100% weight; in Figure 13, the weighing combination in the top corner gives 100% weight to “Carbon Emission” and 0% to “Compressive Strength” and “Cost Efficiency”. The 0% weight line for this parameter is located opposite each corner. Any point on the triangle’s base gives “Carbon Emission” 0% of its weight, and the weights are distributed from “100% Compressive strength/0% Cost Efficiency” in the bottom-left corner to “0% Compressive strength/100% Cost Efficiency” in the bottom-right corner.

The weighting combinations for the selected parameters of all mortars were identified and placed in the triangle. If each parameter is given equal weight, the minimal value of weighting factors could be set at approx. 30%. Given that all mortars with compressive strength weights of more than 20% meet the class 5 criterion and can thus be utilized for structural applications, the lower limit value for this parameter can be reduced to 20%. As a result, an additional internal performance triangle was formed, reflecting an envelope for acceptable mortar mixtures that satisfy all the requirements. Due to their poor mechanical performance, the blended mortars FA1-50, CCA1-50, CWP1-50, FA2-50 and CWP2-50 did not meet the performance threshold. On another note, the reference mortars C2 and C3 failed to meet the criteria for cost efficiency and carbon emission, respectively, as their associated weights fell short of the required value of 30%. All remaining mortar mixtures met the prescribed criteria and can be declared acceptable in terms of all chosen parameters.

## 6. Conclusions

The presented comprehensive study evaluated the efficiency of three waste materials which are abundantly available in Serbia—fly ash, ceramic waste powder and corn cob ash—as environmentally friendly binder materials in cement–lime masonry mortars. The study concentrated on three key aspects: (1) the characterization of waste materials as potential SCMs, (2) the properties of lime–cement masonry mortar blended with SCMs and (3) the sustainability impact assessment of blended mortar. The principal findings of the study are as follows:

- The determination of the chemical composition of FA and finely ground CCA and CWP indicated a relatively high content of amorphous silica, which positively influenced the pozzolanic activity and manifested itself in high activity index values of these SCMs.
- All SCMs, as conventional pozzolanic materials, required more water to ensure that the necessary workability can be attained when used as partial cement replacement materials in masonry mortar.
- Considering the attained compressive strength, all blended mortars with mix ratios of 1:1:4 and 1:0.7:4.2 satisfied the criterion for structural application, while the blended mortars produced with the ratio of 1:1:5 met the requirement for non-load-bearing masonry elements.
- Due to the increased w/b, the capillary water absorption of blended mortar rose to a significant extent. Despite this trend, all mixes had capillary water absorption coefficient values within the permissible range for the W2 category.
- All mixtures complied with the necessary adhesive strength limit for masonry mortar, exceeding the minimum value of 0.15 MPa.
- The integration of the tested SCMs can lead to a notable reduction in the carbon footprint of the construction process, amounting to approximately 30% less that of the conventional cement–lime mortar. When considering the annual masonry mortar production in the province of Vojvodina, Serbia, the substitution of cement with the specified waste materials presents a substantial opportunity for the mitigation of carbon dioxide emissions, estimated at around 41,500 tons. Additionally, an enhanced cost-efficiency of approximately 25% may be realized in this context. In light of the concept of the performance index and the weighting triangle, the blended mortars CCA2-50, FA3-50, CCA3-50 and CWP3-50 can be deemed to have satisfactory mechanical properties and a favorable environmental influence.

The findings of this study are highly valuable as they allow for the conclusion that an innovative masonry mortar can be efficiently produced without the use of expensive chemical additives and be utilized for structural applications, thus offering a cost-effective and ecologically responsible approach to mitigating the adverse impact of traditional binders on global warming. The other significant properties of masonry mortars, such as thermal conductivity, drying shrinkage, frost resistance and the alkali–silica reaction, were not addressed in the current study. These issues will be the subject of further studies.

## Figures and Tables

**Table 1:** Chemical composition of SCMs.

	FA	CCA	CWP
Loss of ignition at 950 °C	1.50	2.40	3.3
SiO_2_, %	53.64	45.76	60.86
Al_2_O_3_, %	25.74	5.91	16.38
Fe_2_O_3_, %	7.36	3.37	6.81
Na_2_O, %	0.30	0.00	0.77
K_2_O, %	1.48	13.10	2.39
MgO, %	3.09	8.30	3.89
CaO, %	7.15	14.08	9.38
SO_3_, %	2.75	1.26	0.80
P_2_O_5_, %	0.06	2.81	0.14
Content Cl^−^, %	<0.01	0.50	0.002
Reactive SiO_2_, %	48.16	38.21	50.26

**Table 2:** Physical properties of SCMs.

	Criteria	Standard	FA	CCA	CWP
Specific gravity (g/cm^3^)	/	EN 196-6	2.046	2.494	2.633
Specific surface area (cm^2^/g)	/	EN 196-6	6159	6834	4809
Fineness (%)	≤12% (S) ≤40% (N)	EN 933-10 EN 450-1	0.80 Category S	1.6 Category S	3.30 Category S
Pozzolanic activity	Class 5 f_cs_ ≥ 5 MPa f_fl_ ≥ 2 MPa Class 10 f_cs_ ≥ 10 MPa f_fl_ ≥ 3 MPa	SRPS B.C1.018	Class 10	Class 5	Class 10
Activity index	AI_28_ ≥ 75% AI_90_ ≥ 85%	EN 450-1	AI_28_ = 96% AI_90_ = 99%	AI_28_ = 101% AI_90_ = 103%	AI_28_ = 93% AI_90_ = 99%
Initial setting time (min)	≥60	EN 196-3 EN 197-1 EN 450-1	245	270	155
Final setting time (min)	≤2 times the setting of the test cement alone	EN 196-3 EN 197-1 EN 450-1	330 ≤ 2 × 210	395 ≤ 2 × 210	235 ≤ 2 × 210
Soundness (mm)	≤10	EN 196-3 EN 450-1	0.2	0.4	0.2

**Table 3:** Labels and component material quantities for designed masonry mortars.

Mortar	m_c_ (g)	m_l_ (g)	m_s_ (g)	m_scm_ (g)	w/b	m_w_ (g)
C1	161.4	74	1350	/	1.15	270.7
FA1-50	80.7	74	1350	49.6	1.35	275.9
CCA1-50	80.7	74	1350	57.3	1.30	275.6
CWP1-50	80.7	74	1350	61.8	1.30	281.5
C2	193.7	59.2	1350	/	1.05	265.6
FA2-50	96.9	59.2	1350	59.6	1.25	269.5
CCA2-50	96.9	59.2	1350	68.7	1.18	265.2
CWP2-50	96.9	59.2	1350	74.2	1.20	276.3
C3	201.8	92.5	1350	/	0.90	264.9
FA3-50	100.9	92.5	1350	62.0	1.05	268.2
CCA3-50	100.9	92.5	1350	71.6	1.00	265.0
CWP3-50	100.9	92.5	1350	77.3	1.00	270.7

m_c_—mass of cement; m_l_—mass of lime; m_s_—mass of sand; m_scm_—mass of SCM; m_w_—mass of water; w/b–water to binder ratio.

**Table 4:** Class of masonry mortars based on the achieved compressive strength.

	C1	FA1-50	CCA1-50	CWP1-50	C2	FA2-50	CCA2-50	CWP2-50	C3	FA3-50	CCA3-50	CWP3-50
Compressive strength (MPa)	7.97	3.70	4.11	3.18	11.46	5.89	6.41	5.10	13.18	6.35	7.55	6.09
CLASS	5	2.5	2.5	2.5	10	5	5	5	10	5	5	5

**Table 5:** Water-to-binder ratio and the effective water-to-binder ratio.

	C1	FA1-50	CCA1-50	CWP1-50	C2	FA2-50	CCA2-50	CWP2-50	C3	FA3-50	CCA3-50	CWP3-50
w/b	1.15	1.35	1.30	1.30	1.05	1.25	1.18	1.20	0.90	1.05	1.00	1.00
Effective w/b	1.15	1.71	1.70	1.73	1.05	1.64	1.61	1.67	0.90	1.33	1.31	1.33

**Table 6:** Fracture patterns of mortars.

	C1	FA1-50	CCA1-50	CWP1-50	C2	FA2-50	CCA2-50	CWP2-50	C3	FA3-50	CCA3-50	CWP3-50
Pattern	a	a	a	a	b	b	b	b	a	a	b	a

**Table 7:** Water vapor permeability (Wvp) and water vapor resistance factor (μ).

Mortar	Water Vapor Permeability		Water Vapor Resistance Factor	
	Wvp (kg/m·s·Pa)	ΔWvp (%)	μ	Δμ (%)
C1	5.37·10^−11^ ± 3.48·10^−12^	0.00	3.63 ± 0.236	0
FA1-50	4.05·10^−11^ ± 3.29·10^−12^	−24.53	4.82 ± 0.370	32.77
CCA1-50	4.88·10^−11^ ± 3.55·10^−12^	−9.22	4.00 ± 0.291	10.28
CWP1-50	4.36·10^−11^ ± 2.40·10^−12^	−18.85	4.46 ± 0.236	23.07

**Table 8:** Emission factors of materials by weight.

Material	Sand	PC	L	FA	CCA	CWP
CO_2_ (kg/t)	1.4	860	760	8	1 *	1 *

* Unit values were used for the analysis because CO_2_ emission is considered to be negligible during the shredding and grinding processes.

**Table 9:** The unit costs of raw materials.

Material	Sand	PC	L	W	FA	CCA	CWP
Price (EUR/t)	18	170	130	2	1.8	1	1

**Table 10:** The performance indexes of mortar mixtures.

Mortar	Properties						Performance Index						
	Compressive Strength (MPa)	Flexural Strength (MPa)	Capillary w. Absorption (kg/m^2^ × min^0,5^)	Adhesion (MPa)	Carbon Emission (kg/t)	Cost Efficiency (EUR/t)	Compressive Strength	Flexural Strength	Capillary w. Absorption	Adhesion	Carbon Emission	Cost Efficiency	ΣPI
C1	7.97	2.46	0.07	0.75	256.85	80.61	0.60	0.64	0.67	0.79	0.65	0.78	**4.13**
FA1-50	3.70	1.67	0.12	0.60	166.99	62.87	0.28	0.44	0.38	0.64	1.00	1.00	**3.73**
CCA1-50	4.11	1.09	0.13	0.43	**166.54**	**62.83**	0.31	0.29	0.33	0.46	1.00	1.00	**3.39**
CWP1-50	3.18	0.86	0.13	0.67	166.58	62.87	0.24	0.23	0.34	0.71	1.00	1.00	**3.51**
C2	11.46	3.20	0.05	0.53	278.29	85.23	0.87	0.84	0.94	0.56	0.60	0.74	**4.55**
FA2-50	5.89	1.91	0.09	0.85	170.47	63.94	0.45	0.50	0.49	0.90	0.98	0.98	**4.30**
CCA2-50	6.41	1.38	0.09	0.91	169.94	63.88	0.49	0.36	0.50	0.95	0.98	0.98	**4.26**
CWP2-50	5.10	1.54	0.11	0.62	169.96	63.92	0.39	0.40	0.39	0.65	0.98	0.98	**3.80**
C3	**13.18**	**3.82**	**0.05**	0.78	315.28	91.20	1.00	1.00	1.00	0.82	0.53	0.69	**5.03**
FA3-50	6.35	2.30	0.09	**0.95**	204.77	69.38	0.48	0.60	0.48	1.00	0.81	0.91	**4.28**
CCA3-50	7.55	2.29	0.07	0.85	204.22	69.32	0.57	0.60	0.69	0.90	0.82	0.91	**4.48**
CWP3-50	6.09	2.15	0.10	0.79	204.24	69.34	0.46	0.56	0.46	0.83	0.82	0.91	**4.04**

**Table 11:** The weighing factors.

Mortar	Performance Index			Weighing Factors (%)		
	Compressive Strength	Carbon Emission	Cost Efficiency	Compressive Strength	Carbon Emission	Cost Efficiency
C1	0.60	0.65	0.78	29.75	31.90	38.35
FA1-50	0.28	1.00	1.00	12.32	43.80	43.88
CCA1-50	0.31	1.00	1.00	13.71	43.91	43.91
CWP1-50	0.24	1.00	1.00	10.59	43.90	43.88
C2	0.87	0.60	0.74	39.43	27.14	33.43
FA2-50	0.45	0.98	0.98	18.56	40.60	40.84
CCA2-50	0.49	0.98	0.98	19.85	40.00	40.15
CWP2-50	0.39	0.98	0.98	16.48	41.69	41.83
C3	1.00	0.53	0.69	45.25	23.53	31.22
FA3-50	0.48	0.81	0.91	21.99	36.51	41.50
CCA3-50	0.57	0.82	0.91	25.07	35.12	39.81
CWP3-50	0.46	0.82	0.91	21.36	37.07	41.57

## Data Availability

Not applicable.
